# Novel multi-targeted polymerase chain reaction for diagnosis of presumed tubercular uveitis

**DOI:** 10.1186/1869-5760-3-25

**Published:** 2013-01-28

**Authors:** Kusum Sharma, Vishali Gupta, Reema Bansal, Aman Sharma, Meera Sharma, Amod Gupta

**Affiliations:** 1 Department of Medical Microbiology, Post Graduate Institute of Medical Education and Research, Chandigarh, 160012, India; 2 Department of Ophthalmology and Advanced Eye Centre, Post Graduate Institute of Medical Education and Research, Chandigarh 160012, India; 3 Department of Internal Medicine, Post Graduate Institute of Medical Education and Research, Chandigarh, 160012, India

**Keywords:** Uveitis, Tubercular, Polymerase chain reaction, Multi-targeted PCR

## Abstract

**Background:**

The objective of this study was to report the use of multi-targeted polymerase chain reaction (PCR) in the diagnosis of presumed tubercular uveitis. Multi-targeted PCR using three targets specific for *Mycobacterium tuberculosis*, i.e., *IS6110*, *MPB64*, and *protein b*, was performed on intraocular fluid samples of 25 subjects. Nine had presumed tubercular uveitis, six had intraocular inflammation secondary to a nontubercular etiology (disease controls), and ten had no evidence of intraocular inflammation (normal controls). As described previously, response to antitubercular therapy was considered as the gold standard.

**Results:**

Multi-targeted PCR was positive in seven out of nine patients with presumed tubercular uveitis and negative in all normal and disease controls. The sensitivity and specificity were 77.77% and 100%, respectively. For the diagnosis of presumed tubercular uveitis, multi-targeted PCR had a positive predictive value of 100% and a negative predictive value of 88.88%.

**Conclusion:**

Multi-targeted PCR can be a valuable tool for diagnosing presumed tubercular uveitis.

## Background

Establishing a definitive diagnosis of presumed tubercular uveitis is a challenge as the intraocular specimen is seldom available for histopathological or microbiological evaluation. A low bacterial count amounting to a negative acid-fast bacillus smear examination and a delay of about 4 to 6 weeks in confirming diagnosis by bacterial culture further limit the utility of these tests in uveitis patients of presumed tubercular etiology [1,2]. The newer tests like interferon gamma release assays could not differentiate latent from active infection. Also, the nucleic acid amplification techniques (NAAT) like polymerase chain reaction (PCR) have limited sensitivity [3,4]. Hence, the proposed guidelines for diagnosis of presumed tubercular uveitis are still based on suggestive clinical history, signs, supportive investigations, such as positive tuberculin skin test (TST)/QuantiFERON-TB Gold test or a positive chest X-ray, exclusion of other known etiologies of uveitis, and a favorable response to antitubercular therapy (ATT) [1,3-7]. One of the reasons for poor sensitivity of PCR was the absence of a particular genome in 20% to 40% isolates of *Mycobacterium tuberculosis* (MTB) [8-11]. However, the simultaneous amplification of *IS6110* and *MPB64* in patients with osteoarticular tuberculosis (TB) and *IS6110*, *MPB64*, and *protein b* in patients with TB meningitis (TBM) resulted in enhanced sensitivity of 82.5% and 86.63%, respectively [12,13]. We standardized and validated a multi-targeted PCR using three target genes simultaneously, namely *IS6110*, *MPB64*, and *protein b*, for establishing a diagnosis of TB and hence conducted the study to assess the role of this novel multi-targeted PCR in the diagnosis of presumed tubercular uveitis.

## Methods

From a total of 25 patients, 18 vitreous fluid samples, 6 aqueous fluid samples, and 1 iris biopsy were taken for the study. These 25 patients were divided into three groups:

1. Group 1 included nine patients with presumed tubercular uveitis based on clinical signs and corroborative evidence [1]. The diagnosis of TB was presumed when the patient had

(a) Signs of active uveitis such as cells/flare in the anterior chamber, with or without granulomatous keratic precipitates or iris nodules, iris granuloma, vitreous cells, snowballs, snowbanking, retinal vasculitis with/without perivascular choroiditis scars with/without vitreous hemorrhage or tractional retinal detachment, serpiginous-like choroiditis, choroidal granuloma, or neuroretinitis.

(b) A positive TST according to the CDC recommendations [29] (TST, 10 mm induration or more at 48 to 72 h) or QuantiFERON-TB Gold test, or a positive chest X-ray.

(c) All known causes of infectious uveitis or known noninfectious uveitis syndromes ruled out. Patients already receiving ATT prior to initiating our study were excluded.

2. Group 2 included six patients with evidence of intraocular inflammation. These served as positive disease controls and had the following inclusion criteria:

(a) Evidence of active inflammation in the anterior and/or posterior segment.

(b) A documented negative TST (less than 10 mm of induration) at 48 to 72 h and/or negative QuantiFERON-TB Gold test, and a negative chest X-ray for TB.

(c) No evidence of extraocular manifestation of TB.

(d) Never received ATT.

3. Group 3 included ten patients without any evidence of inflammation in the eye who underwent intraocular surgery for noninflammatory ocular conditions such as cataract surgery or vitreous surgery for retinal detachment, macular hole, etc. This group served as the normal controls. No additional tests were done for this group of patients.

Depending upon the severity of intraocular inflammation in the anterior or posterior segment in groups 1 and 2, aqueous or vitreous fluid samples were obtained for the PCR analysis. In group 3, aqueous or vitreous samples were collected depending upon the intraocular surgery being performed (cataract or vitreous surgery). Hence, of nine patients in group 1, aqueous sample was collected from one patient (chronic anterior uveitis with complicated cataract), vitreous from seven (serpiginous-like choroiditis with scleritis (one), retinal vasculitis (three), choroidal granuloma (one), panuveitis (one), intermediate uveitis (one)), and iris biopsy from one patient (iris granuloma). In group 2, all six patients underwent vitreous fluid sample analysis. In group 3, aqueous and vitreous fluid samples were obtained from five patients each. All samples were subjected to MPCR.

Group 1 patients underwent baseline uveitis investigations that included TST and/or QFT-TB Gold test, chest X-ray, hemogram, erythrocyte sedimentation rate, and *Treponema pallidum* hemagglutination (TPHA) test. When required, as after a negative response to ATT, patient no. 8 (recalcitrant panuveitis with rhegmatogenous retinal detachment) underwent pars plana vitrectomy with internal tamponade. No additional laboratory tests were done. Patient no. 9, after a failed response to ATT and corticosteroids, underwent brain MRI on clinically suspecting primary intraocular lymphoma (PIOL), followed by a chorioretinal biopsy for cytological examination that confirmed the diagnosis of PIOL. In group 2 patients, the investigations were ordered as follows: TST, chest X-ray, and TPHA test in intermediate uveitis (one), which were negative; smear and culture examination of the vitreous sample in postoperative (one) and posttraumatic (one) endophthalmitis; smear and culture evaluation of vitreous, blood, and urine samples in presumed endogenous endophthalmitis (one); and serology for *Toxoplasma* antibodies in toxoplasmic retinochoroiditis (one). The remaining patient with phacoantigenic uveitis did not require investigations specific to uveitis.

Patients in group 3 were included only to serve as normal (disease-free) controls. These patients underwent a detailed ocular examination as a routine protocol prior to surgery. Since there was no evidence of any intraocular inflammation, uveitis-related investigations were not considered in these patients. All patients in group 1 were reviewed by a consultant internist. None in groups 2 and 3 required his consultation.

Prior to enrolment, all patients had undergone a detailed ocular examination including best-corrected visual acuity, intraocular pressure, and slit lamp biomicroscopy. Ancillary tests like fundus photography, fundus fluorescein angiography, spectral domain optical coherence tomography, and ultrasound B scan were performed as and when required. Systemic evaluation was done by a consultant internist (AS) with special interest in systemic inflammatory diseases. This study has been approved by the Institute Ethics Committee.

### Collection of samples

An informed consent was taken from all the study participants. The vitreous humor, aqueous fluid, and/or iris biopsy were collected from the eye with active uveitis (groups 1 and 2) under strict aseptic precautions and transferred to the laboratory immediately.

### Extraction of DNA and running samples for PCR

DNA was extracted from the samples using DNeasy Tissue Kit (QIAGEN, Hilden, Germany) according to the manufacturer's instructions. The extracted DNA specimens were stored at −20°C until used.

### Multi-targeted PCR

Multi-targeted PCR was carried out as described previously [12]. To check the internal reproducibility of the test, it was repeated three times and put up by two people independently. In each independent muti-targeted PCR assay, test results were compared with the results for one positive and one negative control. The positive control included the DNA of H37Rv, and the negative control included PCR-grade water. Identification of MTB was done using a specific pair of primers designed to amplify *IS6110*, *protein antigen b*, and *MPB64* in the MTB complex, and the expected band size was 123 bp for *IS6110*, 419 bp for *protein b*, and 240 bp for *MPB64*. The sequences of primers used for *protein b* PCR were Pab f and Pab r: 5-ACC ACC GAG CGG TTC GCC TGA-3 and 5-GAT CTG CGG GTC GTC CCA GGT-3, respectively. Primers used for *IS6110* were IS1: CCTGCGAGCGTAGGCGT-30 and IS2: 5-CTCGTCCAGCG CCGCTTCGG-3. Primers used for *MPB64* were MPB1: 5-TCC GCT GCC AGT CGT CTT CC-30 and MPB2: 50-GTC CTC GCG AGT CTA GGC CA-3. The following components were added to the Eppendorf tube (for a 50-μl reaction): PCR buffer (10X), dNTPs (Mix,10 mM), primer IS1 (10 pm/μl), IS2 (10 pm/μl), P1 (10 pm/μl), P2 (10 pm/μl), MPB1 (10 pm/μl), MPB2 (10 pm/μl), Taq polymerase (5 U/μl), DNA template, and water. DNA amplification was performed for 40 cycles following an initial denaturation step at 94°C for 5 min in a thermocycler using the following program: denaturation at 94°C for 1 min, annealing at 65°C for 1.5 min, extension at 72°C for 1.5 min, and final extension at 72°C for 10 min.

The amplified product was stored at 4°C until detection on 1.5% agarose gel stained with ethidium bromide. The stained gel was examined under UV light to look for bands 123 bp for *IS6110*, 419 bp for *protein b*, and 240 bp for *MPB64* using a molecular weight marker of 100-bp ladder. The samples showing the presence of these bands under ultraviolet transillumination were considered positive.

### Specificity and sensitivity of the MPCR assay

In agreement with Eisenach et al. [30], muti-targeted PCR was highly specific for MTB in a preliminary study. No amplification product was produced with other *Mycobacterium* species, such as *M. avium*, *M. fortuitum*, or *M. kansasii* (data not shown). Sensitivity was estimated by serial dilutions of MTB DNA. The MPCR detected 10 fg, which is equivalent to two mycobacterial genomes.

### Multi-targeted PCR quality control

To avoid contamination during DNA extraction and amplification, strict precautions were taken, including separate areas for DNA extraction, reagent preparation, amplification and product detection, and regular meticulous cleaning of surfaces with 10% hypochlorite. In addition, all the reagents were aliquoted upon arrival in the laboratory. Positive and negative controls were included with each set of reaction. The positive control was DNA extracted from H37RV, whereas the negative control was PCR-grade water. To demonstrate the presence of inhibitors in multi-targeted PCR, all negative samples were spiked with positive control DNA, and no inhibitors were detected on spiked samples as all were positive with spiked DNA.

### Statistical methods

The sensitivity, specificity, positive predictive value, and negative predictive value were calculated using standard formulae as follows (positive test: *a* = disease, *b* = no disease; negative test: *c* = disease, *d* = no disease):

• Sensitivity = *a* / (*a* + *c*).

• Specificity = *d* / (*b* + *d*).

• Positive predictive value = *a* / (*a* + *b*).

• Negative predictive value = *d* / (*c* + *d*).

## Results

Group 1 comprised of nine patients with six males and three females. The mean age was 42.5 ± 9.8 years (range 30 to 50 years). Group 2 (six positive controls) had five males and one female. The mean age was 42 ± 20.8 years (range 4 to 60 years). Group 3 (ten negative controls) had five males and five females. The mean age was 47.9 ± 19.8 years (range 5 to 70 years).

The clinical diagnosis in group 1 included vasculitis with or without vitreous hemorrhage/tractional retinal detachment (three), panuveitis (one), serpiginous-like choroiditis (one), choroidal granuloma (one), intermediate uveitis (one), chronic anterior uveitis (one), and iris granuloma (one). Table 1 shows the details of clinical signs in groups 1 and 2.

**Table 1:** Clinical ocular details of patients in groups 1 and 2 who underwent multi-targeted PCR analysis

**Patient no.**	**Group 1**		**Group 2**	
	**Diagnosis**	**Clinical signs**	**Diagnosis**	**Clinical signs**
1	RE serpiginous-like choroiditis	Serpiginous-like choroiditis	RE phacoantigenic uveitis	AC cells ++, flare ++, retained lens cortical matter
2	LE choroidal granuloma	AC cells +, vitreous cells ++, choroidal granuloma	RE postoperative endophthalmitis	Hypopyon, fibrin, vitreous exudates
3	BE retinal vasculitis	RE vitreous cells ++, rhegmatogenous RD; LE vitreous hemorrhage	BE intermediate uveitis	Vitreous cells ++, snowballs ++, CME ++
4	RE iris granuloma	Iris granuloma, AC cells ++, flare +	RE endogenous endophthalmitis	AC cells ++, flare +, vitreous cells +++, exudates ++
5	LE retinal vasculitis	Retinal vasculitis, vitreous hemorrhage, tractional RD	RE toxoplasmic retinochoroiditis	Vitreous cells ++, retinochoroiditis
6	BE chronic anterior uveitis	Cells ++, flare ++, complicated cataract, seclusiopupillae	LE posttraumatic endophthalmitis	Hypopyon, flare +++, cataract, vitreous exudates ++
7	RE intermediate uveitis	Vitreous cells +++, snowballs	-	-
8	BE panuveitis	RE AC cells ++, flare ++, seclusiopupillae, rhegmatogenous RD; LE AC cells +, vitreous cells ++, snowballs ++, CME +	-	-
9	BE retinal vasculitis	Vitreous cells +++, vasculitis	-	-

RE, right eye; LE, left eye; BE, both eyes; AC, anterior chamber; RD, retinal detachment; CME, cystoid macular edema.

**Figure 1:**
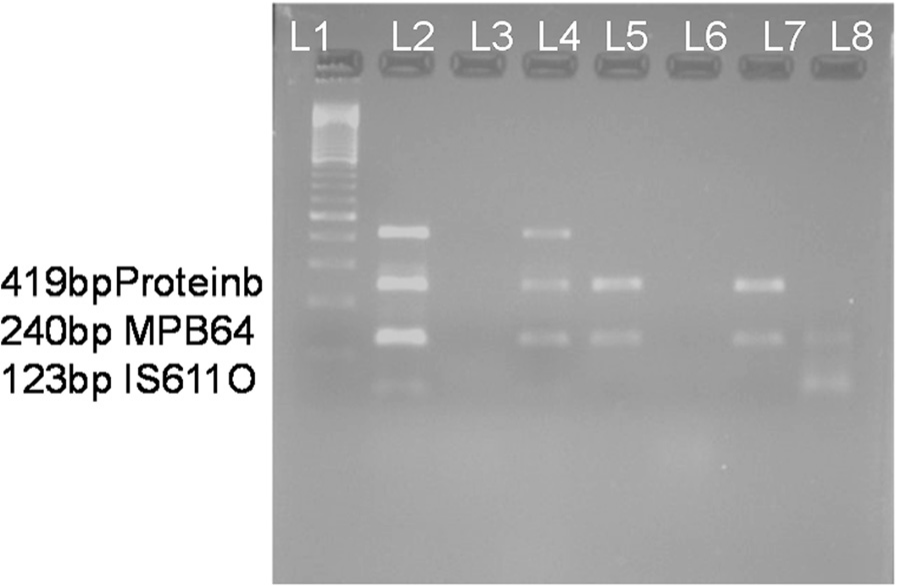
**Results of multi-targeted PCR.** Lane 1 - 100-bp MM; lane 2 - positive control showing all three bands, i.e., 123-bp *IS6110*, 240-bp *MPB64*, and 419-bp *protein b*; lane 3-negative control; lane 4 - positive clinical sample by all three bands; lane 5 and lane 7 - clinical sample positive by *IS6110* and *MPB64* bands; lane 6 - negative clinical sample; lane 8 - clinical sample only *IS6110* positive.

Of nine patients with presumed tubercular uveitis (group 1), seven (77.77%) were positive by multi-targeted PCR (Figure 1). Of these seven multi-targeted PCR-positive cases, all three bands (*IS6110*, *protein b*, and *MPB64*) were present in two cases, and two bands (*MPB64* and *IS6110*) were present in two cases, and a single band each for *MPB64* and *IS6110* was present in two cases and one case, respectively (Table 2). Sensitivity of *IS6110* and *MPB64* was 55.55% and 66.66%, respectively. By using all the three primers, sensitivity of multi-targeted PCR was higher (77.77%). Since multi-targeted PCR was negative in groups 2 and 3 subjects, the specificity was 100%. Overall sensitivity of multi-targeted PCR, *MPB64*, *IS6110*, and *protein b* PCR were 77.77%, 66.66%, 55.55%, and 22.22%, respectively (Table 3). Multi-targeted PCR had a positive predictive value of 100% and a negative predictive value of 88.88%.

**Table 2:** Detailed genome results of positive MPCR in seven of nine cases of presumed tubercular uveitis

**Specific genome results (group 1)**	**Number of samples**
*IS6110* positive alone	1
*MPB64* positive alone	2
Both *MPB64* and *IS6110* positive	2
All three positive (*MPB64*, *IS6110*, and *protein b*)	2

**Table 3:** Sensitivity and specificity of MPCR in presumed tubercular uveitis patients and disease and normal controls

**Test**	**Test results**	**Group 1**	**Groups 2 and 3**	**Sensitivity**	**Specificity**	**PPV**	**NPV**
		**(*n*** **= 9)**	**(*n*** **= 16)**	**(%)**	**(%)**	**(%)**	**(%)**
MPCR	Positive	7	-				
	Negative	2	16	77.77	100	100	88.88
*MPB64*	Positive	6	-				
	Negative	3	16	66.66	100	100	84.21
*IS6110*	Positive	5	-				
	Negative	4	16	55.55	100	100	80.00
*Protein b*	Positive	2	-				
	Negative	7	16	22.22	100	100	69.56

PPV, positive predictive value; NPV, negative predictive value.

The median follow-up of seven patients with a positive multi-targeted PCR in group 1 was 18 months (range 1.5 to 28 months). All patients were started on ATT with corticosteroids (oral/topical). Six of these patients were quiescent at the final visit. One developed recurrence of anterior segment inflammation (cell 2+, flare 2+) during follow-up. Of the other two cases that were negative by multi-targeted PCR, one (patient no. 8) presented with right eye retinal vasculitis with rhegmatogenous retinal detachment and left eye intermediate uveitis. The TST was positive. The patient underwent pars plana vitrectomy with silicon oil tamponade in the right eye and received ATT with oral corticosteroids. The left eye showed persistent inflammation despite treatment at 19 months of follow-up. The other case (patient no. 9) presented with diffuse retinal vasculitis in the right eye and had a positive TST but did not respond to oral corticosteroids and ATT. The patient developed discrete hypopigmented lesions in the fundus that were below the retinal pigment epithelium as revealed by spectral domain optical coherence tomography. Following a negative vitreous biopsy report and other relevant investigations, the patient underwent a repeat vitreous surgery for obtaining retinal and subretinal biopsy that revealed an intraocular B-cell lymphoma.

## Discussion

The clinical diagnosis of presumed tubercular uveitis is challenging due to protean manifestations, especially in an endemic country and BCG-vaccinated population. The chronic, recurrent nature of the disease demands an early and accurate diagnosis since a highly effective ATT is available for treating these patients. The NAAT have emerged as important tools for a rapid and an accurate diagnosis of TB. A meta-analysis of NAAT used in the diagnosis of TB concluded that commercial tests yielded results with high specificity but low sensitivity especially in smear-negative cases, while heterogeneity and low diagnostic accuracy were a concern with in-house PCR tests [14].

For the diagnosis of MTB, a large number of different sequences of the *Mycobacterium* genome have been targeted [15-17]. Most of the NAAT-based diagnostic methods have used a single target (e.g., *IS6110*) for the amplification and detection of tuberculosis [18]. Since *IS6110* is absent in 10% to 40% of MTB isolates especially in geographically endemic areas like India, the likelihood of false-negative tests is increased [10,11,18]. A low sensitivity (37.7%) has been reported with PCR in ocular samples from presumed tubercular uveitis patients [6]. An alternative approach is to use multi-targeted PCR, in which several target genes are amplified simultaneously. This improves the sensitivity, specificity, and rapidity of diagnosis, as seen in pulmonary [19,20] and extrapulmonary TB [12,13,21]. In the present study, though small, the sensitivity of multi-targeted PCR was 77.7%. We have developed and validated multi-targeted PCR (MPCR) using three different targets, i.e., *IS6110*, *MPB64*, and *protein antigen b* (*Pab*). To check the internal reproducibility of the test, it was repeated three times and put up by two people independently.

In the present study, we evaluated MPCR using three different targets, i.e., *IS6110*, *MPB64*, and *Pab* specific for MTB. *IS6110* was chosen because of multiple copy numbers of *IS6110* (6 to 24) in the MTB genome, making it an attractive target for PCR amplification [5]. *MBP64* and *protein b* primers had shown good sensitivity for detection of MTB in our previous experience for the diagnosis of TBM patients [12].

For the diagnosis of tubercular uveitis, most of the previous studies had used only *IS6110*. The sensitivity of PCR is reported between 37% and 58.82% [6,7]. In the present study, sensitivity of *IS6110* was 55.55%, which is comparable to that of previously reported studies [6,7]. The sensitivity of *MPB64* was 66.66%, and the specificity was 100%. An important finding was the presence of two cases, which were only *MPB64* positive but *IS6110* negative. In the absence of *MPB64* primers, these cases would have tested negative for MTB. Similarly, one case that was only *IS6110* positive would have tested MTB negative by using only *MPB64* primers. *MPB64* as a target for diagnosis of tubercular uveitis has been evaluated in 23 patients suspected of Eales' disease and 27 control group subjects by Madhavan et al. and had shown a sensitivity of 47.8% and a specificity of 88.8% [22]. The authors mentioned that *MPB64* is 10,000 times more sensitive than *IS6110* for the diagnosis of tubercular uveitis.

*Protein b* PCR was positive in two out of nine patients in group 1. *Protein b* is a 38-kDa protein which is specific for MTB complex. This genome has not been evaluated for diagnosis of tubercular uveitis previously. It did not enhance the sensitivity of MPCR in the present study but had a good specificity for diagnosis of TBM in a previous study [23] and needs to be evaluated in a larger number of patients in future prospective studies for diagnosis of tubercular uveitis.

Multiple gene amplification studies for the diagnosis of tubercular uveitis have not been reported, although a few studies have evaluated multiple targets as separate uniplex PCRs in other extrapulmonary conditions. These have not been studied as multi-targeted PCR and had reported increase in sensitivity by using two targets together [24]. This multi-targeted PCR was validated in culture-positive sputum samples in another laboratory (unpublished data). In our previous report of patients with TBM who underwent multi-targeted PCR analysis, the sensitivity of multi-targeted PCR was 94.4% in confirmed cases of TBM and 84.4% in suspected cases of TBM, and the specificity was 100% [12]. In the present study, sensitivity and specificity of multi-targeted PCR for diagnosis of presumed tubercular uveitis were 77.7% and 100%, respectively. Two of group 1 patients had a negative MPCR. A possible reason for a negative result by multi-targeted PCR in patient 8 could be due to a low number of bacteria or poor lysis of bacteria in the ocular fluids. The tough cell wall of bacteria also can make the isolation of DNA difficult [25]. It could also be affected by the presence of inhibitors in the samples [26]. However, since patient no. 8 showed a poor response to ATT and oral corticosteroids, a nontubercular etiology or drug resistance cannot be ruled out. Patient no. 9 with a negative MPCR had primary intraocular B-cell lymphoma that was diagnosed from a subretinal biopsy following a poor response to ATT and oral corticosteroids. If we exclude the case of B-cell lymphoma (patient no. 9) from group 1, the sensitivity of multi-targeted PCR increases to 90%. To the best of our knowledge, this is the first attempt to describe the use of novel multi-targeted PCR for diagnosing presumed tubercular uveitis.

Presence of TB DNA in the ocular fluid suggests the presence of *Mycobacterium* infection within the eye but does not differentiate the clinical picture from an immune response to mycobacterial antigens being loaded from the retinal pigment epithelium (RPE) cells, a possible sanctuary for MTB, or from somewhere else in the body. Demonstration of several acid-fast bacteria localized within the necrotic RPE cells in an earlier report by Rao et al. supports the possibility of tubercular uveitis as an immune response to these sequestered bacteria within the RPE cells [27]. DNA-based PCR cannot differentiate between active and dormant infection. In a latent infection of the eye, a healthy and disease control group patient may also have a positive multi-targeted PCR, but this needs to be studied in a larger number of patients. The advantage of this study is that due to the inherent paucibacilliary nature of the disease, resulting in negligible culture positivity, response to ATT and multi-targeted PCR positivity may be surrogate markers for uveitis of tubercular origin. However, even though a highly effective ATT treatment is available, this treatment is not free of adverse effects and some degree of certainty in the diagnosis of tuberculous uveitis in the form of clinical signs and corroborative laboratory evidence is needed before putting patients on treatment.

We have earlier reported the use of quantitative PCR that allowed a fast detection and quantification of the mycobacterial load in the tested specimens of presumed tubercular uveitis [28]. However, our study has certain limitations, a small number of patients being the major limitation. The gold standard used in our study for diagnosing tubercular uveitis is a subject of major concern and controversy. In the absence of mycobacterial culture as the gold standard for diagnosing TB infection, the next best alternative for labeling these patients as having presumed tubercular uveitis is a favorable response to ATT in addition to the corroborative laboratory evidence, as used by many studies over the years. Also, our control group 3 has potential flaws. These patients were enrolled for cataract or vitreoretinal surgery. In the absence of any evidence of uveitis (active or inactive), these patients were presumed to be normal, and investigations related to uveitis work-up were not considered necessary in them. The follow-up is limited in our patients. Due to weaknesses in the study design, we are not able to arrive to statistically significant conclusions and are just showing preliminary results that would need further study.

Early diagnosis and prompt treatment of presumed tubercular uveitis is required for better patient outcome. Novel multi-targeted PCR using three primers has shown a high sensitivity and specificity for diagnosing presumed tubercular uveitis.

## Conclusions

In resource-limited countries that are endemic for tuberculosis, multi-targeted PCR has a high potential to be used as a diagnostic modality. However, more studies with a large sample size are required to corroborate its utility in a rapid and accurate diagnosis of presumed tubercular uveitis.

## Competing interests

The authors declare that they have no competing interests.

## Authors’ contributions

KS participated in designing the study, carried out the PCR tests, performed the statistical analysis, and wrote the manuscript. VG participated in the patient recruitment and clinical sample collection. RB participated in the patient recruitment, clinical sample collection, patient treatment follow-up, and manuscript writing. AS participated in the study design, clinical diagnosis, statistical analysis, and manuscript writing. MS participated in carrying out the microbiological investigations. AG participated in the overall designing of the study, patient recruitment, clinical sample collection, clinical diagnosis, treatment and follow-up, manuscript writing, and overall supervision of the research project. All authors read and approved the final manuscript.
